# miR1908-5p regulates energy homeostasis in hepatocyte models

**DOI:** 10.1038/s41598-021-03156-4

**Published:** 2021-12-09

**Authors:** Sébastien Soubeyrand, Paulina Lau, Kaitlyn Beehler, Kelsey McShane, Ruth McPherson

**Affiliations:** 1 Atherogenomics Laboratory, University of Ottawa Heart Institute, Ottawa, Canada; 2 Division of Cardiology, Ruddy Canadian Cardiovascular Genetics Centre, University of Ottawa Heart Institute, Ottawa, Canada

**Keywords:** Biochemistry, Cancer, Cell biology, Physiology, Diseases, Molecular medicine, Pathogenesis

## Abstract

We previously identified genomic variants that are quantitative trait loci for circulating miR-1908-5p and then showed this microRNA to causally associate with plasma levels of LDL-C, fasting blood glucose and HbA1c. The link to LDL-C was subsequently validated and clarified by the identification of a miR1908-5p-TGFB-LDLR regulatory axis. Here, we continue our investigations on miR1908-5p function by leveraging human primary hepatocytes and HuH-7 hepatoma models. Expression of miR1908-5p was shown to be sensitive to glucose and agents affecting glucose metabolism. Transcriptome-wide changes in primary hepatocytes and HuH-7 cells treated with a miR1908-5p mimic were investigated by enrichment approaches to identify targeted transcripts and cognate pathways. Significant pathways included autophagy and increased mitochondrial function. Reduced activation and/or levels of several key energy and metabolic regulators (AKT, mTOR, ME1, G6PD, AMPK and LKB) were subsequently confirmed in mimic treated HuH-7 cells. These effects were associated with reduced NADPH to NADP+ ratio in HuH-7 cells. LKB1 was validated as a direct target of miR1908-5p, the reintroduction of which was however insufficient to compensate for the impact of the miR1908-5p mimic on AMPK and ACC1. These findings implicate miR1908-5p in metabolic and energy regulation in hepatocyte models via multiple, independent, pathways.

## Introduction

We recently identified a set of circulating miRNAs as significant QTLs for common genetic variants^1^. miRNAs are short RNAs exhibiting various levels of conservation that regulate all aspects of cellular biology, both physiological and pathological^2^. This is generally achieved through formation of protein/RNA RISC complexes that interact with cognate mRNAs to modulate protein expression^3^. Typically, miRNAs singly regulate multiple targets, thereby helping shape and regulate biological pathways. Due to their exceptional stability, circulating miRNAs have been used as biomarkers for various traits and conditions^2^.

One of particular interest, miR1908-5p, is a primate-specific miRNA that is inversely correlated to plasma LDL-C, and glycemic variables, fasting glucose and glycated hemoglobin (A1c). By 2-Sample Mendelian Randomization analysis, we demonstrated these to be causal associations^1^. Mechanisms linking LDL-C to miR1908-5p were recently clarified when we demonstrated that miR1908-5p promotes LDL receptor function via suppression of Transforming Growth Factor beta and Bone Morphogenic Protein 1^4^. Here we have explored the relationship between miR1908-5p and glycemic variables.

The liver plays a central integrative role in glucose homeostasis by storing glucose as glycogen when blood glucose increases and by synthesizing and releasing it when needed^5^. Long-term suppression of hepatic glucose production by insulin is mediated by AKT/FOXO1 signaling^6^. AKT is also implicated in the AKT/Mammalian target of rapamycin (mTOR) cascade which promotes growth and/or proliferation^7^. Furthermore, the latter axis suppresses the Liver Kinase B1 (LKB1)- 5′AMP-activated protein kinase (AMPK) axis, whose role is to redress low energy states by increasing catabolism and reducing anabolism^8–10^.

In primary hepatocytes and hepatocarcinoma cell lines, we now show that miR1908-5p is linked to several processes, including suppression of two antagonistic pathways (mTOR and AMPK) and a reduction in NADPH/NADP+ levels in HuH-7 cells. Moreover, we identify *LKB1* as a miR1908-5p target in both HuH-7 cells and primary hepatocytes. These findings demonstrate a role for miR1908-5p in the regulation of hepatocyte energy metabolism via the reprogramming of antagonistic pathways.

## Methods

### Human tissue biopsies

Liver and omental adipose biopsies were obtained from consenting healthy obese individuals at the time of bariatric surgery. Samples were flash-frozen in liquid nitrogen and stored at − 80 °C until analysis. Samples were then homogenized in TriPure Isolation Reagent (Roche Diagnostics) and RNA was isolated as described below. The study was carried out in accordance with Helsinki guidelines for human research and was approved by the Research Ethics Board of the Ottawa Hospital. Written informed consent was obtained from all participants.

### Cell culture and treatments

HuH-7 and HepG2 cells were maintained in low-glucose DMEM+ 10% FBS, 1% Penicillin/Streptomycin. For glucose concentration experiments, HuH-7 were switched to no glucose (Gibco, cat#. 11966025) DMEM supplemented with various concentrations of glucose (0.2 g/L, 1 g/L, and 3 g/L). Unless mentioned otherwise, cells were maintained under euglycemic (1 g/l) conditions. HepaRG were grown in William’s E medium with supplements.

Cells were treated with 1 mM AICAR, 2 mM metformin, 10 µM Compound C as indicated. Control cells were cultured with vehicle DMSO (1%). For miRNA transfection, hsa-miR-1908-5p mimic and inhibitor (miRIDIAN), as well as their matching controls, were purchased from Dharmacon. Samples were treated with 10 nM of miRNA in the presence of Lipofectamine RNAiMax (Thermofisher) at a ratio of 3.3 pmol of miRNA per µl of RNAiMax. Unless mentioned otherwise, treatment was for 72 h.

Cryopreserved primary hepatocytes, media and media supplements were obtained from Sigma and ThermoFisher Scientific. Hepatocytes (MTOXH1000 (lot LHuf17912; 32 YO, BMI 23) and HMCPMS (lot HU8360; 37 YO, BMI 32)) were thawed in thawing media and then plated in plating media (Sigma) for 6 h in 8 wells of 12-well plates pre-coated with collagen I. Media changed to culture medium (MED-HHCM; Sigma) which was replaced every 24 h. Replicates (2 for MT0XH100 and 3 for HMCPMS) consisting of 4 treatments each (INH, INHctl, MIM, MIMctl) were performed by staggering transfection by 24 h: the first transfection was performed 72 h after initial plating, the second one was performed 96 h post-plating and the third one (HMCPMS only), 120 h post-plating, respectively. Transfection conditions were identical to HuH-7 (10 nM miRNA final, 3 µl of RNAiMax per 10 pmol miRNA). Cells were washed 3 × in PBS and lysed 72 h post-transfection in TriPure isolation reagent (Roche).

### Western blot

Cells were lysed in IP buffer (150 mM NaCl, 50 mM Tris pH 8, 1% Triton X-100) with Complete Protease Inhibitor and PhoStop Tablets (Roche) followed by centrifugation for 2 min (16,000×*g*) at 4 °C. Proteins were denatured under reducing conditions, resolved on 8% SDS-PAGE gels and transferred for 1 h to nitrocellulose membrane. After blocking for 1 h in Odyssey buffer (LI-COR), the membrane was incubated successively (for antibodies raised in the same species but recognizing proteins whose masses are readily distinguishable post SDS-PAGE ) or jointly (for antibodies raised in different species) in primary antibody (diluted 1:2000 in PBS/0.1% Tween 20) dilutions for 16 h and cognate secondary antibody (Donkey anti-Rabbit 800CW and/or Donkey anti-mouse 680 RD diluted 1:20,000 in PBS/0.1% Tween 20) incubation for 1 h. Washes between incubations were performed using 1 X PBS (4 × 1 min). Acquisition of signals in the 700 and/or 800 channels was performed on a Odyssey Infrared Imager using the Odyssey v3.0 imaging software (LI-COR).

### Reporter assay and LKB1 restoration

To obtain the reporter DNA constructs encompassing either the 3′UTR of LKB1 or a mutated version in which the four predicted miR1908-5p binding sites were deleted, the corresponding 3′UTRs of LKB1 were synthesized in vitro and assembled in pUC57. The resulting fragments were isolated using EcoRI and NgoMIV and were transferred to XbaI linearized pGL3-Promoter (Promega) using HiFi assembly (NEBuilder, New England Biolab). The resulting plasmid designs resulted in the insertion of the 3′UTR between the Luciferase sequence and the SV40 poly(A) signal. See Supplemental Material for complete sequences of the 3′UTR. HuH-7(~ 80% density) in 24 well plates were transfected with miR1908-5p as indicated above, immediately followed by a transfection of either construct supplemented with 2% pRSV-renilla to control for transfection efficiency (Promega). Cells were rinsed briefly 20 h later in PBS and lysed in 0.1 ml of 1 X Passive Lysis Buffer (Promega). Aliquots (10 µl) were assayed for firefly and renilla activity using standard assay conditions and a Glomax luminometer (Promega).

For STK11/LKB1 restoration experiments, HuH-7 cells (24 well plate) treated either with a miR1908 mimic or a control mimic 24 h prior, were transfected with 20 or 100 ng of pHAGE-STK11 (a gift from Gordon Mills & Kenneth Scott; Addgene plasmid # 116794) for an additional 48 h^11^.

### Gene expression and pathway analysis

Cells were lysed using TriPure Isolation Reagent (Roche Diagnostics) and RNA was isolated using the Direct-zol RNA MiniPrep Plus (Zymo Research), including the DNase digestion step, as per supplier's instructions. For miRNA expression, the TaqMan MicroRNA Reverse Transcriptor Kit (Thermofisher) in conjunction with target-specific primers (miR-1908-5p, *RNU48* and/or *miR16*) was used to generate first-strand cDNA, according to manufacturer’s instructions. TaqMan quantification was performed by real-time quantitative PCR on a LightCycler 480 (Roche) using target-specific probes (Thermofisher). miR-1908-5p values were normalized to the average one of 2 housekeeping genes (RNU48 or miR16), based on expression consistency across samples; RNU48 was used for cultured cells and miR16 for biopsies, while mRNA expression was normalized using either *HPRT* and *SRP14* (Fig. 3) or *PPIA* (other figures). Unless mentioned otherwise, gene expression values were normalized to the matching control treatment.

Genome-wide transcription analysis was performed at the Centre for Applied Genomics (TCAG) at the Hospital for Sick Children, Toronto, on three distinct biologics. Purified RNA was converted to cRNAs and hybridized to HuGene-2_0-st transcription array. Results were analyzed by using the Expression and Transcriptome Analysis Consoles (Applied Biosystems). Data is available at the Gene Expression Omnibus (GEO) as GSE164632 (HuH-7) and GSE179931 (primary hepatocytes). For primary hepatocytes, 2 independent repeats using MT0XH1000 and one using HMCPMS were analyzed. Where indicated, qRT-PCR validation was performed using a distinct set of three biologics, using target-specific primers and *PPIA* as a housekeeping transcript. Oligonucleotides used are described in Supplemental Material.

To identify biological patterns consistent with the transcription changes, 3 complementary approaches were used. Over-Representation Analysis (ORA) maps interesting genes within the transcriptome profiles to curated pathways or functional categories (Reactome and Gene Ontology were used) and assesses the statistical significance of the overlap; transcripts undergoing nominally significant changes were used. Gene Set Enrichment analysis involves ranking transcripts according to effect size, regardless of statistical significance; the entire array gene list was subjected to analysis^12^. The analysis allows for activation/inactivation calls, based on effect size and direction. Both analyses were performed via the WebGestalt interface (http://www.webgestalt.org/)^13^. Ingenuity Pathway Analysis (IPA, Qiagen) uses several approaches to identify changes to pathways, regulators etc., consistent with transcriptome changes. Similar to GSEA, the analysis uses a Z score to predict activation (+) or inhibition (−) of the impacted pathways. Ingenuity, Reactome and Gene Ontology gene sets are independently defined.

### ATP assay

ATP in protein lysates was quantified with the ATP Bioluminescence Assay Kit CLSII (Roche). Cells (24 well plates) treated for 72 h with miR1908-5p mimic, inhibitor, or their controls, were washed in PBS prior to the addition of 0.2 ml ice-cold TE (100 mM Tris–HCl, 4 mM EDTA, pH 7.4) and immediately frozen at − 80 °C. Cells were then thawed on ice, scraped, and heated for 7 min @ 96 °C. Samples were then centrifuged (16,000×*g*) at 4 °C for 5 min. The supernatant (1 µl equivalent per well) was used for ATP quantification (100 µl reaction per sample) by luminometry in a GLOMAX (Promega) luminometer (2-s integration per well) using the provided ATP standards to generate standard curves. For cell content normalization, protein in the matching lysate supernatant (40 µl) was measured by Bradford assay.

### NADPH/NADP+ assay

NADPH and NADP+ were quantified enzymatically using fluorescein-based chemistry, essentially as suggested by the supplier (Promega). Briefly, cells seeded in 24 well plates were rinsed 3 X in PBS, topped up with 100 µl of PBS and lysed by the addition of 100 µl of 0.2% NaOH, 1% Dodecyltrimethylammonium bromide (DTAB) for 5 min on ice. Lysates were then heated at 60 °C for 15 min either after the addition of 0.4 N HCl (to isolate NADP+) or as is (to isolate NADPH). All samples were then adjusted to identical buffer conditions using 0.5 M TRIS (+ /- 0.2 N HCl) and NADP(H) levels were measured enzymatically for 30 min and 60 min (with similar findings) in a Glomax luminometer (Promega). Quantification per dinucleotide was performed on the equivalent of 12.5% of total lysate recovered.

### Statistical analysis

Statistical significance was determined via ANOVA or two-tailed t-tests, as appropriate. Tests were performed in Prism.

## Results

### Expression of miR1908 in human liver and omental adipose tissue

**Figure 1:**
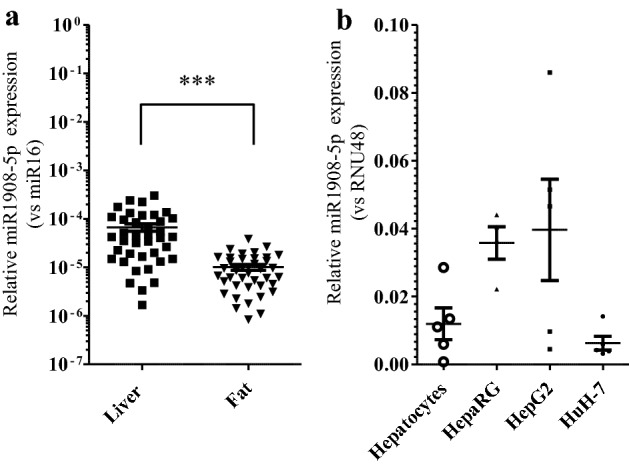
miR1908-5p abundance in hepatocyte models and human biopsies. (**a**) miR1908-5p expression in liver and omental fat biopsies. The difference between adipose and liver was highly significant (*p* < 0.0001, t-test). Values are expressed relative to the average abundance of RNU48 (miR16 was not detectable in primary hepatocytes). (**b**) miR1908-5p expression in hepatocytes and HCCs. Values are expressed relative to the abundance of miR16 (RNU48 detection was inconsistent). Data represent the average of 4–5 biological replicates for HCC (± SEM). Means across cell types were statistically significant (1 way ANOVA, *p* = 0.03), but no pair passed Tukey’s post-hoc multiple comparison test.

Previous analyses demonstrated that circulating miR1908-5p levels are inversely correlated with both fasting blood glucose and A1c and demonstrated this to be a causal relationship^1^. Given the central role of the liver in glucose homeostasis via glucose uptake and production, expression of miR1908-5p was assessed in human liver biopsies. Since miR1908-5p expression was previously linked to adipocyte differentiation and adipocytes play a major role in glucose homeostasis, human omental adipose samples were similarly quantified^14,15^. The analysis revealed expression of miR1908-5p in both tissues, with higher abundance in liver samples (Fig. 1a). Thus, while not excluding extrahepatic functions, the presence of miR1908-5p in the liver is consistent with a hepatic role.

### Expression of miR1908 in hepatocellular carcinoma cells

To identify a model system amenable to further molecular characterization, expression of miR1908-5p was examined in hepatocellular carcinoma (HCC) lines commonly used as proxies to study hepatocyte function. Although primary hepatocytes would represent an ideal choice to dissect the role of miR1908-5p in liver function, they present important limitations, predominantly cost, inter-lot variability and progressive loss of liver markers during ex vivo maintenance. Comparison of 3 available HCC lines (HepG2, HuH-7 and HepaRG) with primary hepatocytes revealed comparable expression of miR1908-5p (Fig. 1b). Of the 3 HCC lines, HuH-7 cells were chosen for most follow-up experiments, since they are readily transfectable, are responsive to glucose, grow well in culture, are capable of gluconeogenesis and express *GLUT2*, although *GLUT1* is overexpressed as is typical in HCC lines^16,17^.

### miR1908 expression is increased by glucose availability and reduced by inhibitors of glycolysis and gluconeogenesis

**Figure 2:**
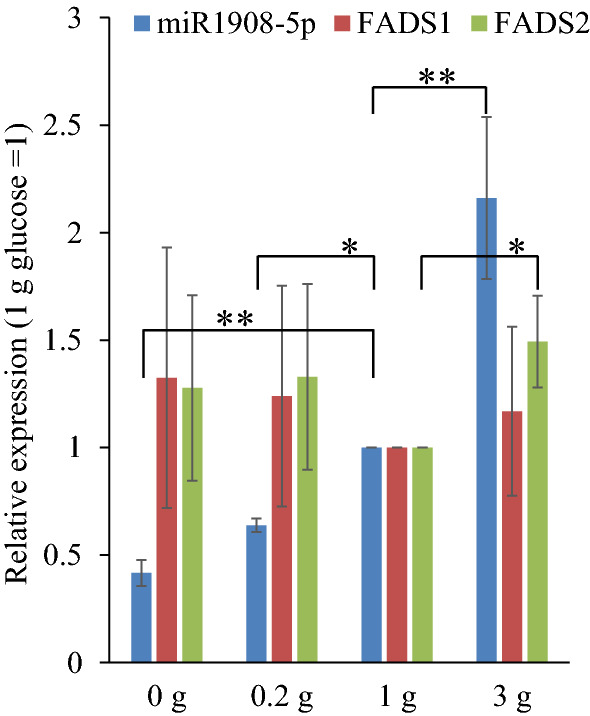
Cellular miR-1908-5p expression is proportional to media glucose concentration in HuH-7 cells. HuH-7 cells were maintained in variable concentrations of glucose (in pyruvate-free media) for 72 h. Cells were lysed, RNA isolated and miR-16, RNU48 and miR1908-5p were quantified by qRT-PCR. Values were normalized using the ΔCp method, using RNU48 and miR1908-5p average value as a reference. Results are expressed relative to the corresponding euglycemic (1 g/L) condition value. The error bars depict the SD. X g/L glucose vs 1 g/L glucose: **p < 0.01; *p < 0.05.

Next, a possible link of miR1908-5p to glucose metabolism was first assessed by exploring the response of miR1908-5p to ambient glucose. HuH-7 were maintained in hypoglycemic, euglycemic and hyperglycemic glucose conditions for 72 h and miR1908-5p levels determined. As shown, miR1908-5p expression was positively correlated with glucose concentration (Fig. 2). By contrast, expression of *FADS1* (an intron of which harbours *MIR1908*) and *FADS2* (which shares a common head-to-head promoter region with *FADS1*), were largely insensitive to glucose concentration. Thus, the *MIR1908* and *FADS1/2* loci appear to be regulated independently, consistent with prior evidence^14,18^.

**Figure 3:**
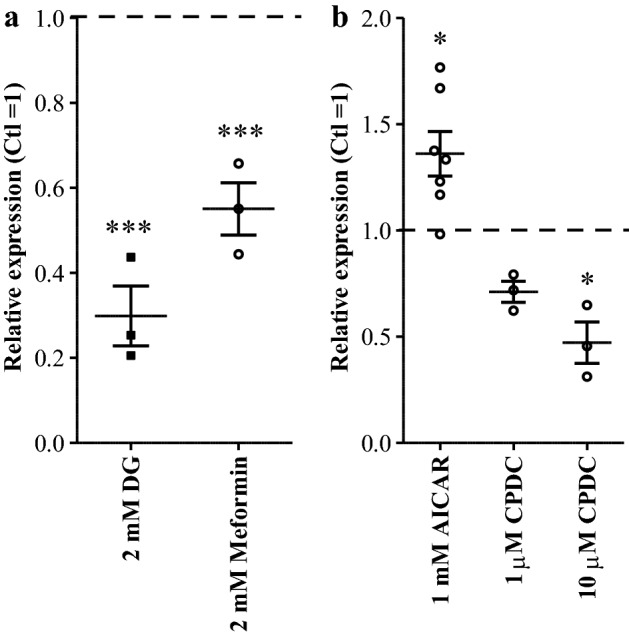
Glucose and energy modulators affect miR1908-5p expression. (**a**) impact of glucose metabolism modulators. (**b**) miR1908-5p expression is sensitive to AMPK regulators. HuH-7 cells were grown in media supplemented with indicated drugs 72 h. RNA was then isolated from lysed cells and miR1908-5p, along miR-16 and RNU48 for normalization, were quantified by qRT-PCR. Normalized values are expressed relative to the vehicle only (Ctl) condition. ANOVA was performed using Dunnett’s post-hoc test. The error bars depict the SEM. *p < 0.05; ***p < 0.001, relative to control.

The role of glucose metabolism in regulating miR1908-5p was examined next. HuH-7 cells were first incubated with the glycolysis inhibitor 2-deoxyglucose (2DG). Prolonged incubation with 2DG reduced miR1908 levels ~ twofold suggesting that ongoing glycolysis is required to sustain miR1908-5p production. Next, cells were treated with metformin, which attenuates liver gluconeogenesis and fatty acid synthesis^19–21^. Metformin was found to reduce miR1908-5p levels (Fig. 3a). Cognizant of the fact that metformin has pleiotropic effects, these findings suggest that impairment of glucose metabolism reduces miR1908-5p expression^22–24^. AMPK is a major metabolic regulator and a major target of metformin’s action^21,25^. A contribution of AMPK to the maintenance of miR1908 expression was assessed by stimulating AMPK activity with the ribonucleotide analog AICAR. Chronic AICAR treatment (1 mM) resulted in increased miR1908-5p levels (Fig. 3b). Conversely, compound C, a relatively non-specific inhibitor of AMPK, reduced miR1908-5p level indicating that AMPK or a related kinase acts to sustain miR1908-5p expression. These findings are in accord with a previous study reporting contrasting effects of metformin and AMPK activators on glycolysis and gluconeogenesis^26^.

### Transcriptome-wide analysis of primary human hepatocytes and HuH-7 cells treated with a miR1908-5p mimic

To gain insight into the pathways regulated by miR1908-5p, transcriptome-wide changes in response to a miR1908-5p mimic and control were examined in primary hepatocytes via microarray analysis. 49 FDR significant transcripts were identified, 45 of which were suppressed by mimic treatment (Table S1). To reveal coherent patterns in the transcription changes, three independent pathway enrichment analyses were performed (See Methods for additional information). First, Over Representation Analysis (ORA) was performed on nominally affected transcripts, using Reactome and Gene Ontology (GO) gene sets (Table S2). Whereas no Reactome pathways passed a strict FDR significance threshold, several GO gene sets associated with a broad range of biological processes passed FDR significance, including “Cellular Response to Stress” and “Response to Growth Factor”. To complement and enrich the ORA analysis, Gene Set Enrichment Analyses (GSEA) were performed, leveraging Reactome and GO curated gene sets. Several FDR significant gene sets were identified (Table S3). Of particular interest in the context of glucose and energy regulation, changes consistent with increased mitochondrial function were identified: cristae formation, respiratory electron transport and mitochondrial respiratory chain complex assembly. Finally, Ingenuity Pathway Analysis (IPA) was performed, leveraging transcripts undergoing > 30% change (< 1.3- and > 1.3-fold) to identify Ingenuity curated canonical pathways. This analysis identified 171 FDR significant canonical pathways (Table S4). The top hit (by FDR significance) was (reduced) “Autophagy” consistent with altered energy homeostasis and reduced AMPK activity.

To identify a model system amenable to mechanistic investigation, HuH-7 cells were also treated with miR1908-5p mimic. In addition, an inhibitor arm was included (datasets publicly available as GSE164632). The analysis resulted in 1341 and 2190 nominally significant IDs for the inhibitor and mimic set, respectively but only 121 IDs were impacted in both sets (see Table S5 and Discussion). Comparison with the mimic data from primary hepatocytes was more consistent: a subset of 392 transcripts, representing 10.5% of hepatocytes and 17.9% of HuH-7 significant (*p* < 0.05) hits, were similarly altered in both cell types (Table S6). Those shared genes which were directionally coherent (293 out of 317), were then subjected to ORA using both Reactome and GO (Biological_Process) reference sets, with the rationale that 1) these transcripts likely represent higher confidence targets and 2) that they may point to hepatocyte pathways that are conserved in HuH-7. The analysis identified several nominally significant Reactome pathways including top hits AMPK/ChREBP, mTOR and TGFB, although stringent FDR significance was not reached (Table S7). GO mapping indicated FDR significant enrichment of 44 gene sets, with the most strongly associated sets linked to biosynthetic processes (Table S8). Importantly, as effect size and direction are not considered in ORA analysis, no conclusion about pathway activation or inhibition can be drawn.

### Reduced expression of NADPH oxidases and NADPH to NADP ratio in response to a miR1908-5p mimic

**Figure 4:**
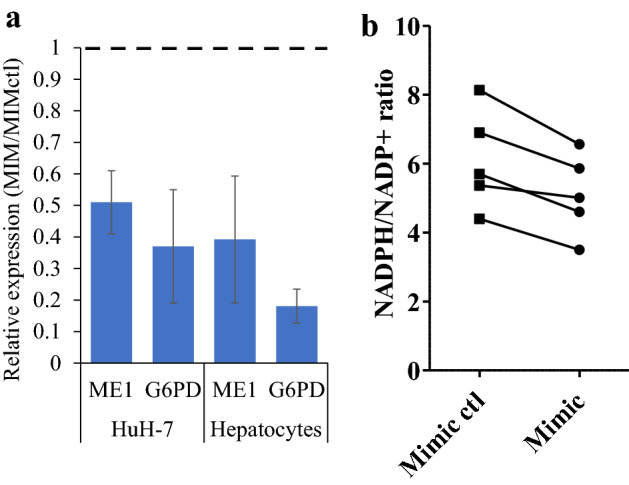
miR1908-5p mimic reduces ME1, G6PD and the NADPH/NADP+ ratio. Lysates from HuH-7 cells and primary hepatocytes treated with miR1908-5p or control were assayed for the presence of **a,** G6PD, ME1 by qRT-PCR or **b**, NADPH and NADP+. In (**a**) MIM vs MIMctl differences were all statistically significant (Student’s t-test < 0.05). In (**b**) Matched samples from each biologic are linked by a line. Treatment with miR1908-5p was associated with a reduction in the ratio of NADPH/NADP+ (0.84 ± 0.06; *p* = 0.007, paired t-test).

The above findings pointed to changes consistent with altered TGFB signaling as well as energy regulation (e.g., autophagy, AMPK, mitochondria). Measurement of cellular ATP following miR1908-5p mimic and inhibitor treatment was performed to assess a possible impact on global energy status. No significant changes were observed, suggesting that energy levels were largely unperturbed (Supplementary Fig. S1). However, examination of shared HuH-7 and primary hepatocyte nominally affected transcripts for ones implicated in energy regulation revealed suppression of two key enzymes involved in the synthesis of NADPH from NADP+, *ME1* and *G6PD* (Table S6). ME1 catalyzes NADP+ to NADPH formation in the cytosol, coupled to the generation of pyruvate from malate whereas *G6PD* is the rate-limiting enzyme bridging glycolysis to the Pentose Phosphate Pathway (PPP), which is important in NADPH, fatty acid, and nucleotide precursors synthesis. Reduced expression of both enzymes was confirmed by qPCR in HuH-7 as well in primary hepatocytes (Fig. 4a).

Lower expression of these two key NAPDH oxidases suggests reduced ability to generate NAPDH from NADP+, possibly leading to reduced steady-state NADPH to NADP+ ratios. This was substantiated by enzymatic assays, demonstrating a ~ 15% diminution in the NADPH to NADP+ ratio in miR1908-5p mimic treated HuH-7 cells (Fig. 4b). By reducing NADPH, which is essential for de novo FA synthesis and glucose utilization through the PPP pathway, miR1908-5p may not only interfere with FA synthesis but also favour glycolysis.

### Suppression of LKB1 expression in response to miR1908 mimic treatment

**Figure 5:**
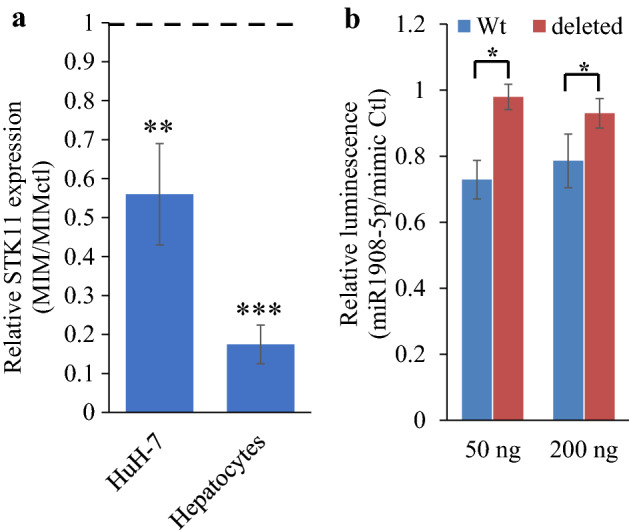
*STK11* is a target of miR1908-5p in HuH-7 and hepatocytes. (**a**) Reduced *STK11* in HuH-7 and Hepatocytes treated with miR1908-5p or control miRNA for 72 h. RNA was isolated and analyzed by qPCR. Expression was internally normalized to PPIA and results are shown as a ratio of the miR1908-5p treated values divided by the miRNA control values. Control values in HuH-7 were 8.7-fold higher than the Hepatocyte values (*p* = 0.0014). Quantification was performed for 3 and 5 independent experiments for HuH-7 and primary hepatocytes, respectively. Error bars represent the 95% C.I. **p < 0.01; ***p < 0.001; MIM vs MIMctl). Dashed line highlights the control value of 1. (**b**) Reporter assay evaluating the contribution of the *STK11* 3′UTR, in the presence of MIM or MIMctl**.** HuH-7 cells were sequentially transfected with miR1908-5p (or mimic ctl) and the indicated amounts of pGL3-PromoterLKB-UTR reporter constructs, either Wt or deleted of putative miR19018-5p binding sites. Quantification of 3 biological replicates (average ± S.D), normalized for each experiment to the mimic control values is shown. Deleted samples values were not statistically significantly different between the miR1908-5p and the mimic control. *p < 0.05 between Wt and deleted constructs.

Bioinformatic identification of altered AMPK signaling and evidence of AMPK implication in miR1908-5p regulation by pharmacological approaches prompted us to examine the hepatocyte and HuH-7 transcriptome datasets, looking for individual transcripts that might connect miR1908-5p to AMPK. Interestingly, mimic treatment resulted in a significant reduction of *STK11*/*LKB1* a key activator of AMPK in both cell models, which was confirmed by qPCR (Fig. 5a)^8,27^. Moreover, IPA predicted reduced AMPK signaling (q = 0.003) (Table S4).

TargetScan (http://www.targetscan.org/vert_72/) identified four potential binding sites (two 8mer and two 7mer-m8) in the *LKB1* 3′UT; *STK11/LKB1* ranked 11th out of 2522 putative transcript targets based on Cumulative Weighted Score (Table S9). To experimentally validate *LKB1* as a *bona fide* target, the 3′UTR, either wild-type or with the four binding sites deleted, was inserted in a reporter construct and introduced in HuH-7 cells, in the presence of either miR1908-5p mimic or control. Luciferase activity was reduced in the presence of the wild-type sequence, but not in the deletion, in a miR1908-5p dependent manner, thus demonstrating that the 3′UTR of *LKB1* is a miR1908-5p target (Fig. 5b).

### miR1908-5p reduces LKB1 levels and AMPK activity in HuH-7 cells

**Figure 6:**
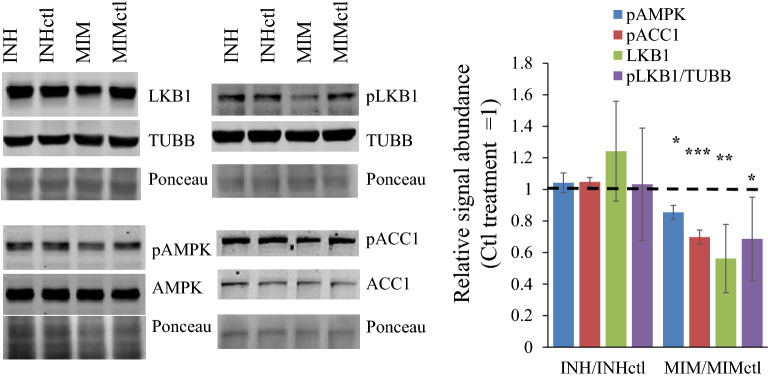
miR1908-5p mimic transfection reduces AMPK activity, LKB1 levels and ACC phosphorylation in HuH-7 cells. HuH-7 cells were treated for 72 h with either mimic inhibitor or miR1908 mimic, and their matching controls. Three distinct gels were probed; Ponceau of the 50 kDa region as loading control is shown. Quantification (average ± S.D from 3 biologics) is expressed relative to unphosphorylated (AMPK, ACC1) or TUBB (LKB1) and normalized internally to the matching control value MIM or INH vs matching ctl: *p < 0.05; **p < 0.01;***p < 0.001.

Next, the impact of *LKB1* suppression was assessed at the protein level, by measuring LKB1 abundance by Western blotting in miR1908-5p and inhibitor-treated HuH-7 cells. While inhibitor treatment resulted in a trend (1.2-fold, *p* = 0.11) towards increased LKB1, mimic treatment was accompanied by a ~ 50% reduction in LKB1 protein abundance, consistent with the *LKB1* findings (Fig. 6). Moreover, LKB1 phosphorylation at S428 which was shown to be involved in LKB1 activation, at least under some conditions, was also reduced^28,29^. In view of the crucial role of LKB1 in promoting AMPK activation via Thr172 phosphorylation of AMPKα, phosphorylation was examined next. In agreement with reduced LKB1 and pLKB1 levels, miR1908-5p treatment significantly reduced Thr172 phosphorylation (Fig. 6). To ascertain that reduced AMPK phosphorylation had functional consequences, we determined the effects on inhibitory phosphorylation of Acetyl-CoA Carboxylase 1 (ACC1), a well-characterized AMPK substrate. Consistent with lower AMPK activity, ACC1 phosphorylation was reduced. Thus, while maintaining miR1908-5p expression requires AMPK, these findings suggest the presence of a negative feedback loop whereby miR1908-5p inhibits AMPK, via LKB1 targeting.

### Reduced LKB1 is insufficient to account for the impact of miR1908-5p on AMPK and ACC1

**Figure 7:**
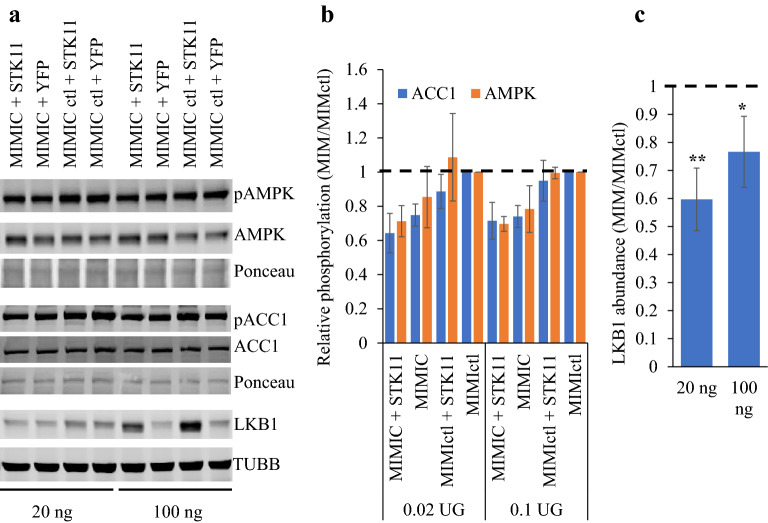
Exogenous LKB1 is insufficient for AMPK phosphorylation recovery and is sensitive to miR1908-5p. HuH-7 cells (24 well plate) treated either with a miR1908 mimic or a control mimic were transfected with 20 or 100 ng of pHAGE-STK11 (or PLVX-YFP) and analyzed by western blot with the indicated antibodies. Ponceau shown represent the regions matching the AMPK (50–60 kDa) and ACC1 (250 kDa) signals. (**a**) representative Western blots (3 distinct gels, with matching Ponceau or TUBB signal). (**b**) quantification of relative phosphorylation from 2 independent determinations, normalized to the MIMIC ctl value (± S.D.); 20 ng plasmid resulted in a 12% LKB1 increase while 100 ng yielded a 3.2-fold increase (over matching YFP control). (**c**) recombinant LKB1 is affected by miR1908-5p. LKB1 levels were measured relative to TUBB in 4 distinct experiments performed as in (**b**). Bars represent the relative LKB1 abundance, expressed as the MIMIC LKB1/TUBB value over the corresponding MIMICctl value. MIM vs MIMctl: *p < 0.05; **p < 0.01.

To evaluate the role of LKB1 suppression in reduced AMPK signaling, miR1908-5p treated or control cells were transfected with pHAGE-*LKB*. Surprisingly, *LKB1* overexpression failed to restore AMPK or ACC1 phosphorylation, suggesting that further changes, secondary to miR1908-5p introduction, are dominant (Fig. 7a,b). Moreover, the transfected *LKB1*, which has neither native 5′ nor 3′ UTRs, was sensitive to miR1908-5p (Fig. 7c). These findings suggest that LKB1 levels are controlled by miR1908-5p only partially through *LKB1* 3′UTR and point to additional effects of miR1908-5p that ultimately converge to control both LKB1 abundance and signaling.

### Suppression of the PI3K/AKT/mTOR axis by miR1908-5p

**Figure 8:**
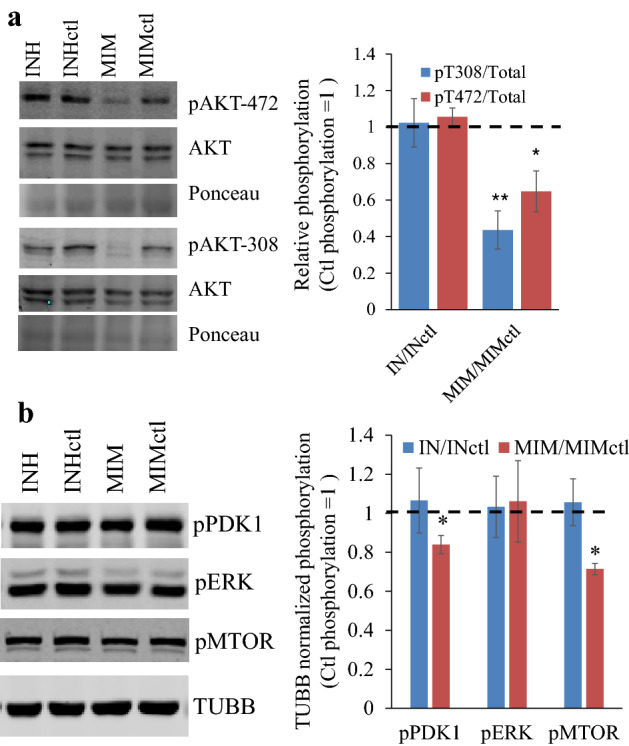
Suppression of the PI3K/AKT/mTOR axis by miR1908-5p. Western blot analyses of HuH-7 cells treated for 72 h with either Mimic inhibitor or miR1908-5p mimic, or their matching controls. (**a**) AKT phosphorylation. Ponceau of the 50 kDa region as loading control is shown. Representative Western blots (2 distinct blots) on the left. Right, the ratio of phospho to non-phospho signal normalized to the matching miRNA (mimic or inhibitor control) value, from 3 biological replicates (average ± S.D). (**b**) PDK1, ERK and MTOR phosphorylation. Representative Western blot on the left (single reprobed blot). Right, quantification of 3 biological replicates (average ± S.D), relative to the TUBB signal and normalized to the matching control oligo value. MIM or INH vs matching ctl: *p < 0.05; **p < 0.01.

The PI3K/AKT/mTOR axis typically acts counter to AMPK signaling and could account for reduced AMPK activity. C-terminal phosphorylation of AMPK by AKT is inhibitory and prevents AMPKα Thr172 phosphorylation^30^. Given that the PI3K/AKT/mTOR axis is frequently activated in cancer cells to sustain proliferation and survival, we examined the status of AKT in response to miR1908 targeting^31^. AKT activation requires at least 2 important and independently regulated phosphorylation events at T308 (via PDPK1 and/or MAPK4), within the activation loop of AKT and S473 (mTORC2) within its hydrophobic loop^32,33^. Of the 2 events, T308 phosphorylation more closely reflects its activity^34^. miR1908-5p mimic treatment elicited a pronounced suppression of phosphorylation on both residues (Fig. 8). Activities of PDK1 and mTOR were measured using phosphorylation levels as proxies of enzyme activity. Significantly miR1908-5p transfection was associated with reduced phospho (active) PDK1 and mTOR, relative to the housekeeper tubulin (Fig. 8). By contrast, pERK previously reported to undergo compensatory phosphorylation upon AKT inhibition in HCC, was unaffected^35^.

## Discussion

**Figure 9:**
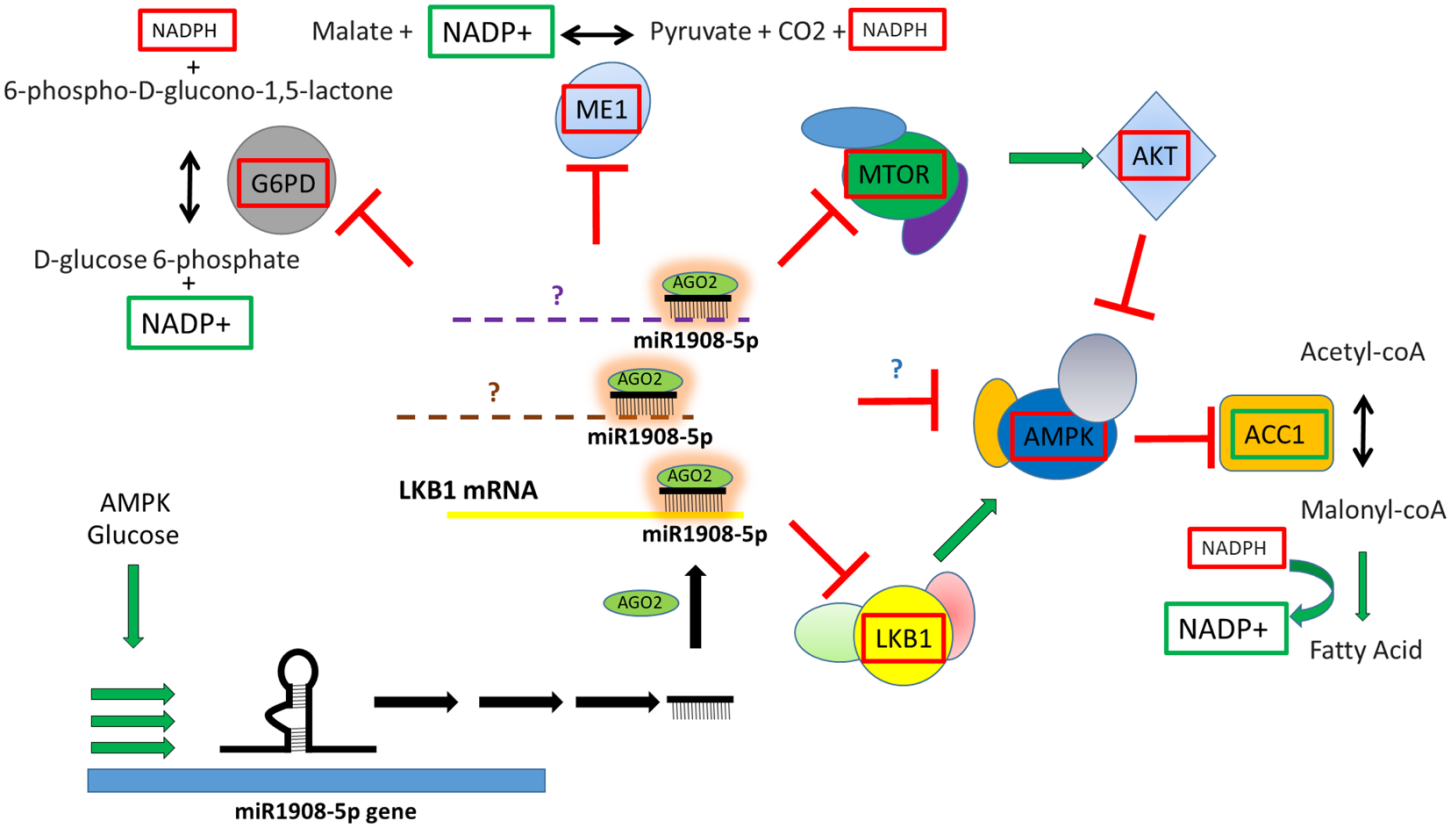
miR1908-5p interacts with cognate target mRNA to regulate the cellular energy landscape. miR1908-5p affects energy (NADP) homeostasis and sensing. miR1908-5p abundance is under the influence of glucose abundance and AMPK activity. In turn, miR1908-5p reduces LKB1 and AMPK levels and activity, in part via LKB1 transcript inhibition, while inhibiting mTOR and AKT. This is correlated with reduced ACC1 phosphorylation which is predicted to increase its activity and promote fatty acid synthesis. Increased fatty acid synthesis may however be counteracted by reduced NADPH availability due to lower G6PD and ME1 expression. Red and green highlights indicate reduced or increased activation and/or level, respectively as a result of miR1908-5p action.

Here we have investigated the biology underlying the negative and causal association between miR1908-5p and glycemic traits. In a series of experiments, we have determined the effects of manipulating miR1908-5p expression and action in both normal and transformed hepatocytes, as summarized in Fig. 9. As expected, given the mechanisms of action of miRNAs as network modulators, miR1908-5p mimic treatment impacted a broad range of transcripts in primary hepatocytes and HuH-7 hepatoma models, as highlighted by the diversity of FDR significant pathways identified. Interestingly, both ORA and IPA implicate TGFB signaling (GO:0007179 and “TGF-β Signaling”) in both primary hepatocytes and HuH-7 cells (Tables S4 and S8). These findings are consistent with our prior work demonstrating reduced *TGFB* abundance in response to mimic treatment^4^. Uniquely, GSEA identified increased mitochondria function (TCA cycle, respiratory electron cycle etc.). The importance of mitochondria might have been missed by IPA since it carries only 2 canonical pathways related to mitochondria (mitochondria dysfunction (170 members) and L-carnitine shuttle pathway (18 members), neither of which was significantly affected. The pathways identified should be helpful in steering future investigations of miR1908-5p and liver function, both normal and pathological.

In addition, miR1908-5p mimic transfection led to a lower abundance of two important regulators of NADPH metabolism, *ME1* and *G6PD*, associated with reduced NADPH/NADP+ ratio in HuH-7 cells. The consequences of reduced NADPH/NADP+ are potentially wide-ranging given the importance of NADPH as a reducing agent and as an indispensable cofactor in anabolic reactions^36,37^. The relative contribution of NADPH regenerating enzymes (G6PD, ME1, ME2, NNT, IDH1 and IDH2) in HuH-7 or liver cells is unknown but we hypothesize that a lower NADPH/NADP+ ratio is due to lower G6PD expression. G6PD is the main contributor of NADPH (from NADP+) in proliferating cells through the Pentose Phosphate Pathway and G6PD targeting leads to reduced NADPH and impaired cell growth in several cancer models^37,38^. G6PD is also essential to the preservation of the redox state in non-cancerous cells, likely through the maintenance of an adequate NADPH supply^39^. We did not however observe an impact on cell growth in HuH-7 cells (AlamarBlue and Mitotracker assays; *data not shown*). Whether this indicates that remaining NADPH/G6PD is sufficient to support normal proliferation, inadequate sensitivity, or the establishment of compensatory adaptations is unclear. Previous studies yielded discrepant results with regard to the effects of miR1908-5p overexpression on cellular proliferation^18,40^.

A limitation of our findings with regard to human liver function is our deliberate focus on HuH-7 cells. Whereas we examined the effect of miR1908-5p manipulation on the transcriptomes of both normal and transformed hepatocytes, protein-centric experiments employed HuH-7 cells, largely for cost, availability, and reproducibility issues. For instance, reduced AKT phosphorylation associated with the introduction of miR1908-5p mimic suggests that the response of HuH-7 to insulin might be impaired by miR1908-5p. Although not examined directly in HuH-7, certain HCC cell lines exhibit impaired gluconeogenesis, high basal AKT phosphorylation and fail to respond adequately to insulin^41^. Whether primary hepatocytes would respond similarly to miR1908-5p remains to be investigated. Moreover, the extra hepatic roles of miR-1908-5p have not been evaluated. Glucose metabolism and energy homeostasis are physiologically determined by a complex interplay between multiple organs. In this regard, the presence of miR1908-5p in adipose tissue as well as in the circulation is consistent with an extra-hepatic function.

In contrast to the AKT findings reported here, *MIR1908* overexpression in neuronal models was shown to activate AKT^42^. Whereas *MIR1908* O/E was linked to *PTEN* suppression in glioblastoma cells, no such relationship was evident in HuH-7, according to the transcription array data. Moreover, as *MIR1908* O/E generates both 5p and 3p forms, which were not analyzed separately, it is possible that miR1908-3p rather than miR1908-5p drives AKT activation. This may underlie the recently reported link of miR1908-3p to breast cancer: miR1908-3p has been identified as a biomarker for breast cancer and shown to promote breast cancer cell proliferation ex vivo^43^. Earlier work suggested that miR1908-3p may be more unstable, consistent with publicly available miRbase data and GEUVADIS data showing ~ 5 to 10 × more reads for miR1908-5p^1,44^.

Unlike mimic treatment, inhibitor treatment did not yield statistically significant changes in HuH-7 cells. This is unlikely to be a consequence of ineffective inhibition as levels of miR1908-5p were routinely reduced by > 95% in response to inhibitor treatment (data not shown). Closer inspection however revealed that inhibitor treatment increased marginally, but systematically, mimic sensitive targets (e.g. Figs. 6, 8). A similar pattern was observed in our prior characterization of *TGFB* in response to miR1908-5p inhibitor^4^. The lack of statistical power may explain why transcriptome analyses of mimic- and inhibitor-treated cells are incoherent: introduction of exogenously sourced miR1908-5p mimic leads to the silencing of miR1908-5p targets that are basally only weakly impacted, perhaps because of their higher basal expression relative to endogenous miR1908-5p. As a result, introduction of the miR1908-5p inhibitor to counter pre-existing miR1908-5p/mRNA complexes may produce subtle changes that oligonucleotide arrays are unable to detect given their limited dynamic range^45^. An important correlate is that the effects of miR1908-5p identified via our mimic approach, while valuable insofar as they inform on the role of miR1908-5p by identifying cognate targets, are likely inflated.

Here, a major finding is that miR1908-5p reduces *LKB1* in HuH-7 and primary hepatocytes. Moreover, the effect appeared to be attenuated in HuH-7, although the difference did not quite reach statistical significance (*p* = 0.07, HuH-7 vs Hepatocytes). This may be attributable to higher basal *LKB1* levels (8.7-fold, HuH-7 vs Hepatocyte miR1908-5p mimic controls, *p* = 0.0017) in HuH-7. LKB1 is crucial to AMPK phosphorylation as its abrogation leads to loss of AMPK phosphorylation and severe hyperglycemia^8^. Significantly, liver *LKB1* ablation was also associated with additional and important expression changes (e.g. strongly upregulated PPGC1-α, ACC1, PKLR etc.). By contrast, no such changes were evident upon miR1908-5p mediated *LKB1* reduction in either system, suggesting that sufficient LKB1 is present to adequately address most cellular demands. Unexpectedly, pHAGE-LKB1 transfection was ineffective at restoring AMPK and ACC1 phosphorylation. We propose that, in line with the integrative properties of miRNAs, miR1908-5p introduces additional dominant changes that reduce LKB1 levels and prevent reconstituted LKB1 from adequately engaging AMPK and ACC1. One possibility involves curtailing or inhibiting other LKB1 regulators. LKB1 is part of a larger trimeric complex with 2 obligate partners (STRAD and MO25) the assembly of which is required for function^27^. While the transcription array data did not identify changes in either transcript, changes at the protein level remain possible.

## Supplementary Information

Supplementary Information 1.

Supplementary Information 2.

## References

[1] Nikpay M (2019). Genome-wide identification of circulating-miRNA expression quantitative trait loci reveals the role of several miRNAs in the regulation of cardiometabolic phenotypes. Cardiovasc. Res..

[2] Gebert LFR, MacRae IJ (2019). Regulation of microRNA function in animals. Nat. Rev. Mol. Cell Biol..

[3] Jonas S, Izaurralde E (2015). Towards a molecular understanding of microRNA-mediated gene silencing. Nat. Rev. Genet..

[4] Beehler K (2021). A Common Polymorphism in the FADS1 Locus Links miR1908 to low-density lipoprotein cholesterol through BMP1. Arterioscler. Thromb. Vasc. Biol..

[5] Petersen MC, Vatner DF, Shulman GI (2017). Regulation of hepatic glucose metabolism in health and disease. Nat. Rev. Endocrinol..

[6] Taniguchi CM (2006). Divergent regulation of hepatic glucose and lipid metabolism by phosphoinositide 3-kinase via Akt and PKCλ/ζ. Cell Metab..

[7] Saxton RA, Sabatini DM (2017). mTOR signaling in growth, metabolism, and disease. Cell.

[8] Shaw RJ (2005). The kinase LKB1 mediates glucose homeostasis in liver and therapeutic effects of metformin. Science (80-.).

[9] Lin SC, Hardie DG (2018). AMPK: Sensing glucose as well as cellular energy status. Cell Metab..

[10] Herzig S, Shaw RJ (2018). AMPK: Guardian of metabolism and mitochondrial homeostasis. Nat. Rev. Mol. Cell Biol..

[11] Nq PKS (2018). Systematic functional annotation of somatic mutations in cancer. Cancer Cell.

[12] Subramanian A (2005). Gene set enrichment analysis: A knowledge-based approach for interpreting genome-wide expression profiles. Proc. Natl. Acad. Sci..

[13] Liao Y, Wang J, Jaehnig EJ, Shi Z, Zhang B (2019). WebGestalt 2019: gene set analysis toolkit with revamped UIs and APIs. Nucl. Acids Res..

[14] Kuang Q (2015). Identification and characterization of NF-kappaB binding sites in human miR-1908 promoter. Biomed. Pharmacother..

[15] Rosen ED, Spiegelman BM (2006). Adipocytes as regulators of energy balance and glucose homeostasis. Nature.

[16] Seyer P (2013). Hepatic glucose sensing is required to preserve β cell glucose competence. J. Clin. Invest..

[17] Gunton JE, Delhanty PJD, Takahashi SI, Baxter RC (2003). Metformin rapidly increases insulin receptor activation in human liver and signals preferentially through insulin-receptor substrate-2. J. Clin. Endocrinol. Metab..

[18] Kim HR (2017). MicroRNA-1908-5p contributes to the oncogenic function of the splicing factor SRSF3. Oncotarget.

[19] Vial G, Detaille D, Guigas B (2019). Role of mitochondria in the mechanism(s) of action of metformin. Front. Endocrinol. (Lausanne).

[20] Yoon JC (2001). Control of hepatic gluconeogenesis through the transcriptional coactivator PGC-1. Nature.

[21] Foretz M (2010). Metformin inhibits hepatic gluconeogenesis in mice independently of the LKB1/AMPK pathway via a decrease in hepatic energy state. J. Clin. Invest..

[22] Miyoshi H (2014). Effect of the anti-diabetic drug metformin in hepatocellular carcinoma in vitro and in vivo. Int. J. Oncol..

[23] Foretz M, Guigas B, Bertrand L, Pollak M, Viollet B (2014). Metformin: From mechanisms of action to therapies. Cell Metab..

[24] Liu X, Romero IL, Litchfield LM, Lengyel E, Locasale JW (2016). Metformin targets central carbon metabolism and reveals mitochondrial requirements in human cancers. Cell Metab..

[25] Wang Y (2019). Metformin improves mitochondrial respiratory activity through activation of AMPK. Cell Rep..

[26] Moonira T (2020). Metformin lowers glucose 6-phosphate in hepatocytes by activation of glycolysis downstream of glucose phosphorylation. J. Biol. Chem..

[27] Zeqiraj E (2009). ATP and MO25α regulate the conformational state of the STRADα pseudokinase and activation of the LKB1 tumour suppressor. PLoS Biol..

[28] Fogarty S, Hardie DG (2009). C-terminal phosphorylation of LKB1 is not required for regulation of AMP-activated protein kinase, BRSK1, BRSK2, or cell cycle arrest. J. Biol. Chem..

[29] Xie Z (2006). Activation of protein kinase C zeta by peroxynitrite regulates LKB1-dependent AMP-activated protein kinase in cultured endothelial cells. J. Biol. Chem..

[30] Hawley SA (2014). Phosphorylation by Akt within the ST loop of AMPK-α1 down-regulates its activation in tumour cells. Biochem. J..

[31] Grabinski N (2012). Combined targeting of AKT and mTOR synergistically inhibits proliferation of hepatocellular carcinoma cells. Mol. Cancer.

[32] Alessi DR (1996). Mechanism of activation of protein kinase B by insulin and IGF-1. EMBO J..

[33] Wang W, Moore DD, Yang F (2019). MAPK4 overexpression promotes tumor progression via noncanonical activation of AKT/mTOR signaling The Journal of Clinical Investigation. J Clin Invest.

[34] Vincent EE (2011). Akt phosphorylation on Thr308 but not on Ser473 correlates with Akt protein kinase activity in human non-small cell lung cancer. Br. J. Cancer.

[35] Ewald F (2015). Vertical targeting of AKT and mTOR as well as dual targeting of AKT and MEK signaling is synergistic in hepatocellular carcinoma. J. Cancer.

[36] Houtkooper RH, Cantó C, Wanders RJ, Auwerx J (2010). The secret life of NAD+: An old metabolite controlling new metabolic signaling pathways. Endocrine Rev..

[37] Ju HQ, Lin JF, Tian T, Xie D, Xu RH (2020). NADPH homeostasis in cancer: functions, mechanisms and therapeutic implications. Signal Trans. Target. Therapy.

[38] Fan J (2014). Quantitative flux analysis reveals folate-dependent NADPH production. Nature.

[39] Leopold JA, Cap A, Scribner AW, Stanton RC, Loscalzo J (2001). Glucose-6-phosphate dehydrogenase deficiency promotes endothelial oxidant stress and decreases endothelial nitric oxide bioavailability. FASEB J..

[40] Li Y, Gan L, Li W, Qin S, Liu G (2019). microRNA-1908-5p inhibits proliferation and promotes apoptosis by targeting PP5 in NSCLC. Int. J. Clin. Exp. Pathol..

[41] Molinaro A, Becattini B, Solinas G (2020). Insulin signaling and glucose metabolism in different hepatoma cell lines deviate from hepatocyte physiology toward a convergent aberrant phenotype. Sci. Rep..

[42] Xia X (2015). MicroRNA-1908 functions as a glioblastoma oncogene by suppressing PTEN tumor suppressor pathway. Mol. Cancer.

[43] Zhu Y (2020). Evaluation of MiR-1908-3p as a novel serum biomarker for breast cancer and analysis its oncogenic function and target genes. BMC Cancer.

[44] Lappalainen T (2013). Transcriptome and genome sequencing uncovers functional variation in humans. Nature.

[45] Xu X (2013). Parallel comparison of Illumina RNA-Seq and Affymetrix microarray platforms on transcriptomic profiles generated from 5-aza-deoxy-cytidine treated HT-29 colon cancer cells and simulated datasets. BMC Bioinform..

