# Supplementary material for: miR1908-5p regulates energy homeostasis in hepatocyte models
Source: Sci Rep. 2021 Dec 9;11:23748. doi: 10.1038/s41598-021-03156-4 (PMC8660805; doi:10.1038/s41598-021-03156-4)

Supplementary information

miR1908-5p regulates energy homeostasis in hepatocyte models

Sébastien Soubeyrand, PhD[1]*, Paulina Lau MSc[1], Kaitlyn Beehler[1], Kelsey McShane[1] and Ruth McPherson, MD, PhD [1,2]*.

Supplementary Figure S1

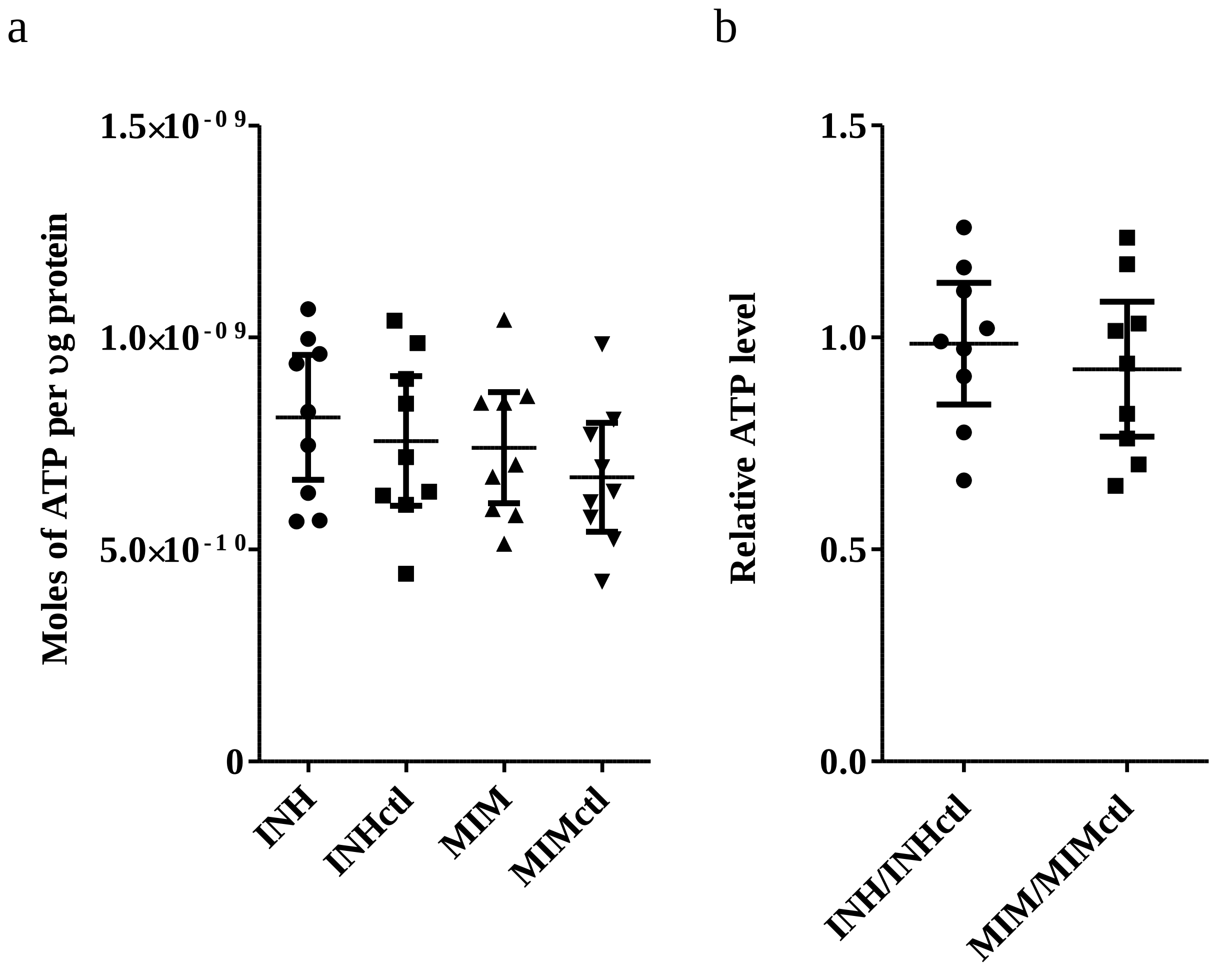

Supplementary Figure S1. Levels of ATP are unaffected upon miR1908-5p inhibitor or mimic treatment.
ATP levels were measured in HuH-7 cells treated for 72 h with miRNA inhibitor, mimic and their matching controls. In a, data is expressed as moles of ATP per ug of protein and represent the average of 9 biologics. In b, bars represent the average value of the relative levels (protein corrected) of ATP in INH or MIM treated samples, relative to their matching control. Error bars represent the 95% Confidence Interval (α =0.05). Differences (INH vs INHctl or MIM vs MIMctl) were not statistically significant.

Supplementary Material

qPCR Primers (Forward and Reverse)

STK11/LKB1

CCACCGAGGTCATCTACCA

GCTGGATGACATTTTTGTGCCG

ME1

TGGAGAGCGTATTCTTGGCT

TGCATTCACATTGGCAAAAT

G6PD

GAGGCCGTGTACACCAAGAT

AATATAGGGGATGGGCTTGG

PPIA

ACCGTGTTCTTCGACATTGC

TTCTGTGAAAGCAGGAACCC

Antibodies used:

ACC1 (Cell Signaling; 3676)

ACC1 pS79 (rndsystems; MAB6898-SP)

AKT (Cell signaling; 2920)

AKT pS473 (Cell signaling; 9271)

AKT pS308 (Cell signaling; 2965)

AMPK Cell signaling (Cell signaling; 2793)

AMPK pT172 (Cell signaling; 2635)

pERK (Cell signaling; 4370)

LKB1 (Cell signaling; 3050)

LKB1 p428 (Cell signaling; 3482)

MTOR pS2478 (Cell signaling;5436)

PDK1 pS241 (Cell signaling; 3438)

TUBB (Genetex; GTX11307)

LKB1 3’UTR contructs

Insert was excised EcoRI/NgoMIV

Vector was linearized with XbaI

Assembly performed with NEBuilder, New England Biolab

WT3UTR

gaattc*Ggaaagatcgccgtgtaatt*GGCTGGCCGCCTGCAGCCCGTGTCCAGGAG**CCCCGCC**AGGTGCC
CGCGCCAGGCCCTCAGTCTTCCTGCCGGTTCCGCCCGCCCTCCCGGAGAGGTGGCCGCCATGCTTCTGTG
CCGACCACGCCCCAGGACCTCCGGAGCGCCCTGCAGGGCCGGGCAGGGGGACAGCAGGGACCGGGCGCAG
CCCTCCCCCCTCGGCCGCCCGGCAGTGCACGCGGCTTGTTGACTTCGCAGCCCCGGGCGGAGCCTTCCCG
GGCGGGCGTGGGAGGAGGGAGGCGGCCTCCATGCACTTTATGTGGAGACTACTGG**CCCCGCC**CGTGGCCT
CGTGCTCCGCAGGGCGCCCAGCGCCGTCCGGCGG**CCCCGCC**GCAGACCAGCTGGCGGGTGTGGAGACCAG
GCTCCTGA**CCCCGCC**ATGCATGCAGCGCCACCTGGAAGCCGCGCGGCCGCTTTGGTTTTTTGTTTGGTTG
GTTCCATTTTCTTTTTTTTCTTTTTTTTTTAAGAAAAAATAAAAGGTGGATTTGAGCTGTGGCTGTGAGG
GGTGTTTGGGAGCTGCTGGGTGGCAGGGGGGCTGTGGGGTCGGGCTCACGTCGCGGCCGCCTTTGCGCTC
TCGGGTCACCCTGCTTTGGCGGCCCGGCCGGAGGGCAGGACCCTCACCTCTCCCCCAAGGCCACTGCGCT
CTTGGGACCCCAGAGAAAACCCGGAGCAAGCAGGAGTGTGCGGTCAATATTTATATCATCCAGAAAAGAA
AAACACGAGAAACGCCATCGCGGGATGGTGCAGACGCGGCGGGGACTCGGAGGGTGCCGTGCGGGCGAGG
CCGCCCAAATTTGGCAATAAATAAAGCTTGGGAAGCTTGGA*ctagagtcggggcggccggccgct*

MUT3UTR

gaattc*Ggaaagatcgccgtgtaatt*GGCTGGCCGCCTGCAGCCCGTGTCCAGGAGAGGTGCCCGCGCCA
GGCCCTCAGTCTTCCTGCCGGTTCCGCCCGCCCTCCCGGAGAGGTGGCCGCCATGCTTCTGTGCCGACCA
CGCCCCAGGACCTCCGGAGCGCCCTGCAGGGCCGGGCAGGGGGACAGCAGGGACCGGGCGCAGCCCTCCC
CCCTCGGCCGCCCGGCAGTGCACGCGGCTTGTTGACTTCGCAGCCCCGGGCGGAGCCTTCCCGGGCGGGC
GTGGGAGGAGGGAGGCGGCCTCCATGCACTTTATGTGGAGACTACTGGCGTGGCCTCGTGCTCCGCAGGG
CGCCCAGCGCCGTCCGGCGGGCAGACCAGCTGGCGGGTGTGGAGACCAGGCTCCTGAATGCATGCAGCGC
CACCTGGAAGCCGCGCGGCCGCTTTGGTTTTTTGTTTGGTTGGTTCCATTTTCTTTTTTTTCTTTTTTTT
TTAAGAAAAAATAAAAGGTGGATTTGAGCTGTGGCTGTGAGGGGTGTTTGGGAGCTGCTGGGTGGCAGGG
GGGCTGTGGGGTCGGGCTCACGTCGCGGCCGCCTTTGCGCTCTCGGGTCACCCTGCTTTGGCGGCCCGGC
CGGAGGGCAGGACCCTCACCTCTCCCCCAAGGCCACTGCGCTCTTGGGACCCCAGAGAAAACCCGGAGCA
AGCAGGAGTGTGCGGTCAATATTTATATCATCCAGAAAAGAAAAACACGAGAAACGCCATCGCGGGATGG
TGCAGACGCGGCGGGGACTCGGAGGGTGCCGTGCGGGCGAGGCCGCCCAAATTTGGCAATAAATAAAGCT
TGGGAAGCTTGGA*ctagagtcggggcggccggccgct*

Fig 6

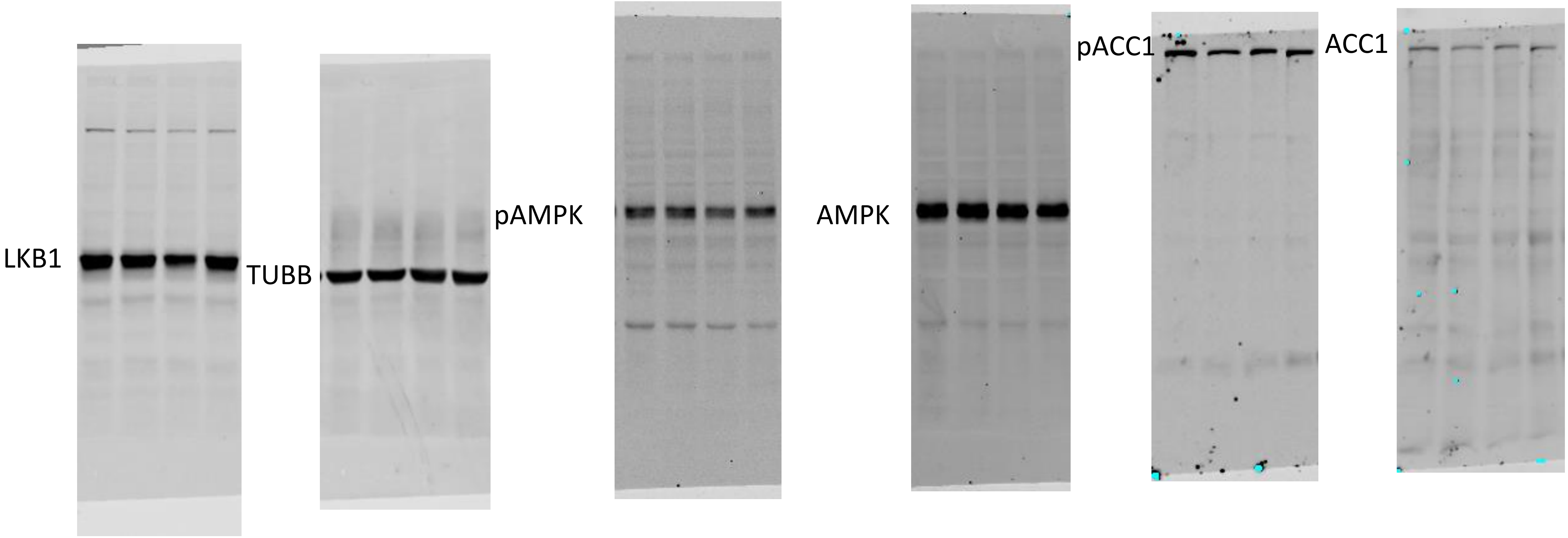

Fig 6 (bis)

TUBB

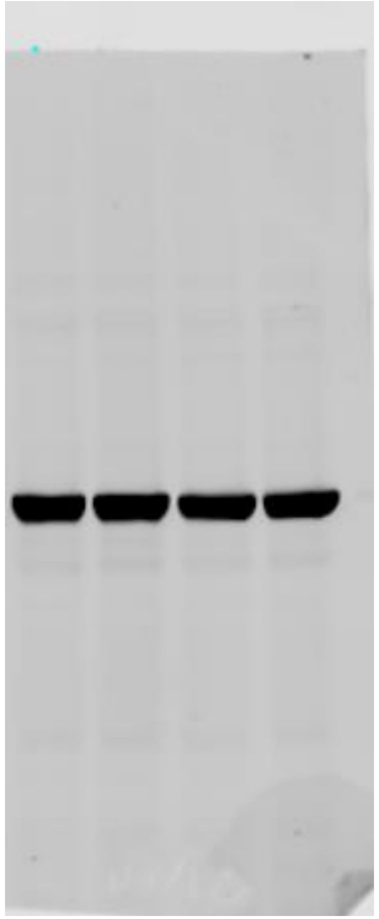

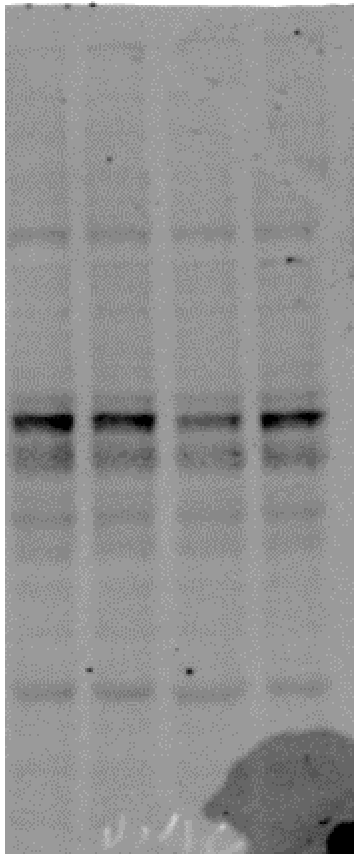

pLKB1

Fig 7

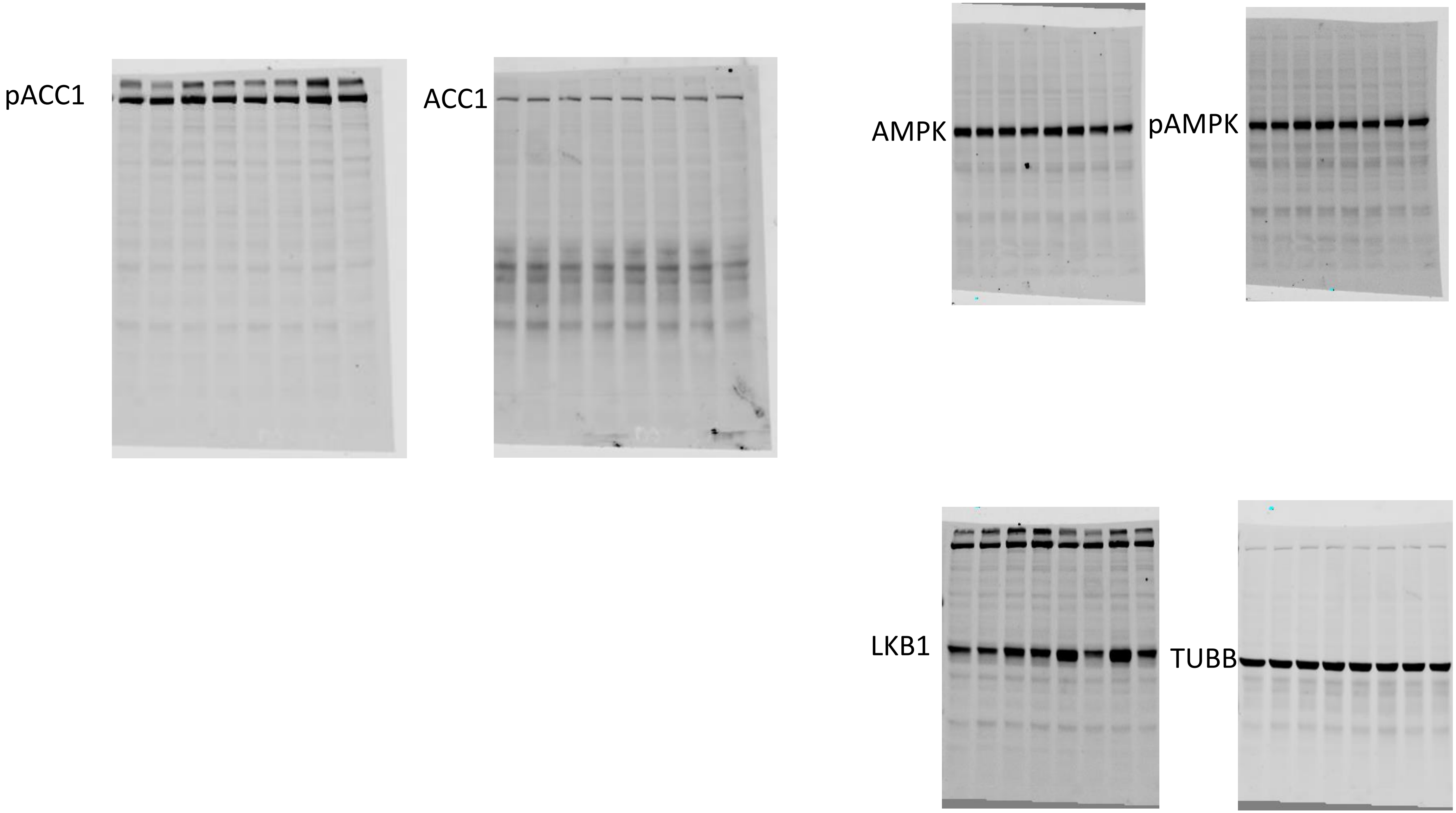

Fig 8a

pAKT 308

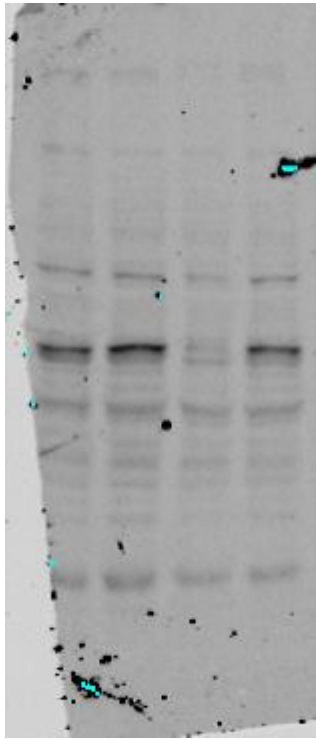

AKT

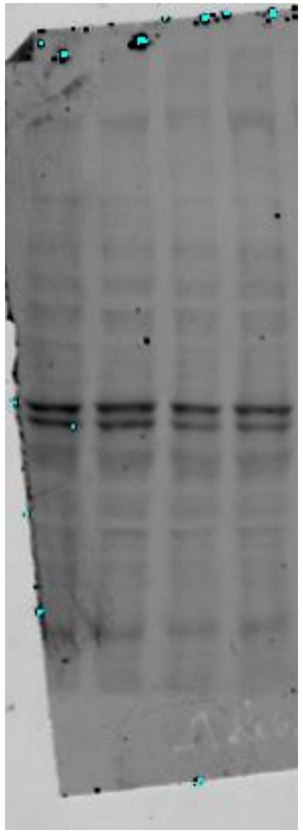

pAKT 472

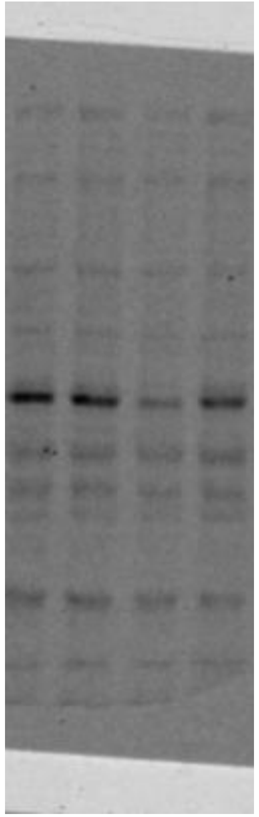

AKT

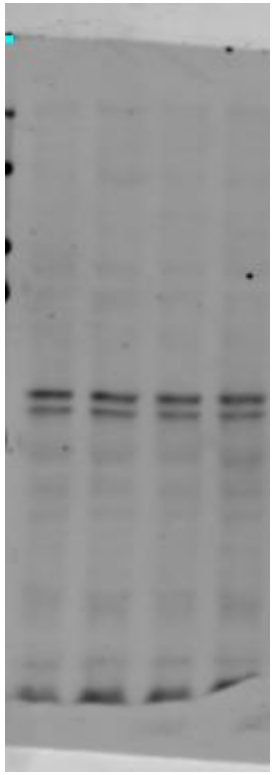

Fig 8b

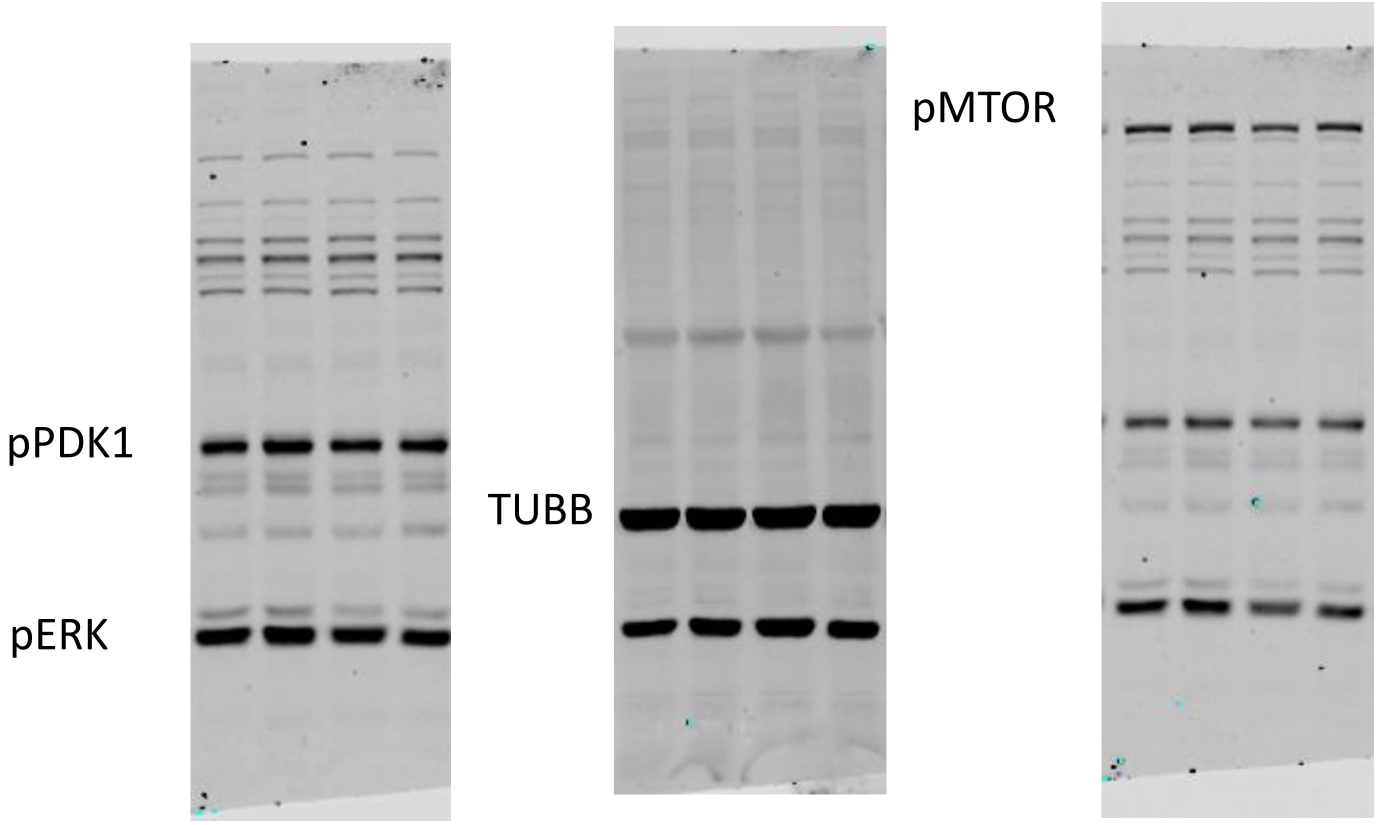

Supplement: Supplementary file 2 — Supplementary Information 2. [file 41598_2021_3156_MOESM2_ESM.pdf]
